# Outcomes of methotrexate therapy for psoriasis and relationship to genetic polymorphisms

**DOI:** 10.1111/j.1365-2133.2008.08898.x

**Published:** 2009-02

**Authors:** RB Warren, RL Smith, E Campalani, S Eyre, CH Smith, JNWN Barker, J Worthington, CEM Griffiths

**Affiliations:** Dermatological Sciences, Salford Royal Hospital, The University of ManchesterManchester M6 8HD, U.K.; * arc Epidemiology Unit, The University of ManchesterManchester M13 9PT, U.K.; † Skin Therapy Research Unit, St John’s Institute of Dermatology, St Thomas’ Hospital, Kings CollegeLondon SE1 7EH, U.K.

**Keywords:** methotrexate, pharmacogenetics, psoriasis

## Abstract

**Background:**

The use of methotrexate is limited by interindividual variability in response. Previous studies in patients with either rheumatoid arthritis or psoriasis suggest that genetic variation across the methotrexate metabolic pathway might enable prediction of both efficacy and toxicity of the drug.

**Objectives:**

To assess if single nucleotide polymorphisms (SNPs) across four genes that are relevant to methotrexate metabolism [folypolyglutamate synthase (*FPGS*), gamma-glutamyl hydrolase (*GGH*), methylenetetrahydrofolate reductase (*MTHFR*) and 5-aminoimidazole-4-carboxamide ribonucleotide transformylase (*ATIC*)] are related to treatment outcomes in patients with psoriasis.

**Methods:**

DNA was collected from 374 patients with psoriasis who had been treated with methotrexate. Data were available on individual outcomes to therapy, namely efficacy and toxicity. Haplotype-tagging SNPs (*r*^2^ > 0·8) for the four genes with a minor allele frequency of > 5% were selected from the HAPMAP phase II data. Genotyping was undertaken using the MassARRAY spectrometric method (Sequenom®).

**Results:**

There were no significant associations detected between clinical outcomes in patients with psoriasis treated with methotrexate and SNPs in the four genes investigated.

**Conclusions:**

Genetic variation in four key genes relevant to the intracellular metabolism of methotrexate does not appear to predict response to methotrexate therapy in patients with psoriasis.

Methotrexate is a first-line systemic therapy for psoriasis; however, its use is limited by unpredictable efficacy and significant hepatotoxicity and gastrointestinal symptoms. Studies in patients with psoriasis and rheumatoid arthritis treated with methotrexate suggest that functional single nucleotide polymorphisms (SNPs) in genes relevant to methotrexate metabolism may influence both efficacy and toxicity of the drug.^1^–^12^ Such studies have focused on isolated functional polymorphisms in only a few genes relevant to methotrexate metabolism and results have been variable, especially in the most investigated gene methylenetetrahydrofolate reductase (*MTHFR*); (Table 1).

**Table 1:** Summary of previous studies in patients with psoriasis and rheumatoid arthritis (RA) of two single nucleotide polymorphisms (C677T**/**rs1801133 and A1298C/rs1801131) in the gene methylenetetrahydrofolate reductase (*MTHFR*) assessing efficacy and toxicity of methotrexate (adapted from Hider *et al*.^13^)

Disease	Polymorphism	No. of patients	Efficacy	Toxicity	Comments	Ref.
Psoriasis	C677T	202	↔	↔	Result for whole cohort, not subgroups	3
RA	C677T	236	↔	↑	All adverse events + abnormal LFT	1
RA	C677T	106	↔	↑		2
RA	C677T	93	↔	↔		4
RA	C677T	531	↔	↔		11
RA	C677T	214	Not reported	↑	CNS adverse event	12
RA	C677T	48	↓	↔		8
RA	C677T	205	↑	↔		5
Psoriasis	A1298C	202	↔	↔	Result for whole cohort, not subgroups	3
RA	A1298C	106	↑	↔		2
RA	A1298C	93	↔	↑		4
RA	A1298C	531	↔	↑		11
RA	A1298C	48	↔	↑		8
RA	A1298C	205	↑	↑		5

↑, Positive association; ↔, neutral finding; ↓, negative association. Some studies reported only associations with specific toxicities such as abnormal liver function tests (LFT) and central nervous system (CNS) effects.

We investigated 47 haplotype-tagging and three functional SNPs in four genes coding for enzymes involved in methotrexate intracellular metabolism: folypolyglutamate synthase (*FPGS*); gamma-glutamyl hydrolase (*GGH*); *MTHFR*; and 5-aminoimidazole-4-carboxamide ribonucleotide transformylase (*ATIC*); (Fig. 1) in a large cohort of patients with psoriasis treated with methotrexate.

**Fig 1:**
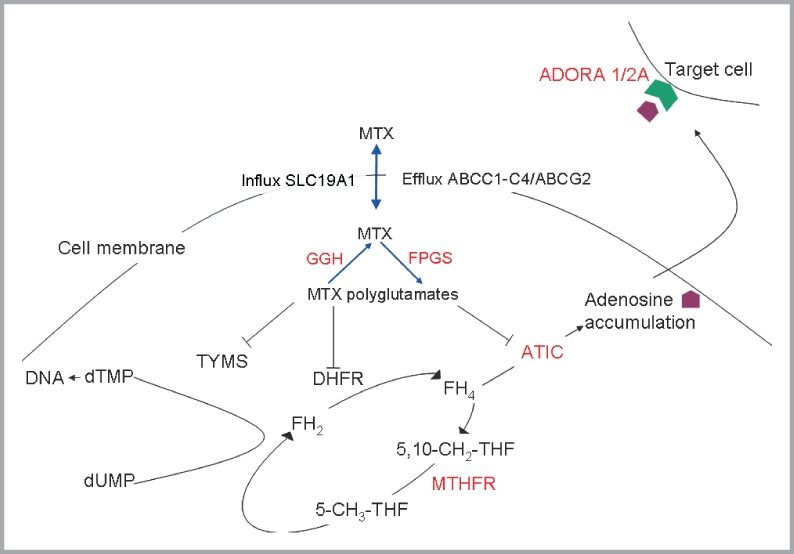
Illustration of some of the key enzymes involved in the metabolism of methotrexate (MTX). MTX is transported into the cell via the solute carrier family 19, member 1 (SLC19A1). It can be actively transported out of the cell by the ATP-binding cassette transporters including ATP-binding cassette, subfamily C (CFTR/MRP), member 1–4 (ABCC1-C4) and ATP-binding cassette, subfamily G, member 2 (ABCG2). Within the cell it undergoes polyglutamation (activation) under the enzymic control of folylpolyglutamate synthase (FPGS). This is a dynamic process where glutamate residues can be removed by gamma-glutamyl hydrolase (GGH). In the polyglutamated form MTX inhibits aminoimidazole-4-carboxamide ribonucleotide transformylase (ATIC), which probably accounts for some of its anti-inflammatory effects via an intracellular rise in adenosine. Inhibition of the folate pathway may not be as important to its mechanism of action in psoriasis, but this pathway includes the enzyme 5,10-methylenetetrahydrofolate reductase (MTHFR) which has been subject to a number of MTX pharmacogenetic studies in the past. MTHFR catalyses the conversion of 5,10-methylenetetrahydrofolate 5,10-CH_2_-THF to 5-methyltetrahydofolate (5-CH_3_-THF), which is a cosubstrate for homocysteine remethylation. The polyglutamated form of MTX also inhibits thymidylate synthase (TYMS), which converts deoxyuridylate (dUMP) to deoxythymidylate (dTMP) in the *de novo* pyrimidine biosynthetic pathway. Genes chosen for this investigation are highlighted in red.

## Methods

This study adheres to the declaration of Helsinki Guidelines and was approved by the relevant local research ethics committees. Subjects provided written, informed consent.

Adult patients who had received methotrexate for treatment of psoriasis were recruited retrospectively from The Dermatology Centre, Salford Royal Hospital, Manchester, and St John’s Institute of Dermatology, London.

### Evaluation of efficacy

Patients were stratified as: (i) ‘responders’––those with clearly documented clinical improvement, using the Psoriasis Area and Severity Index (PASI),^14^ i.e. > 75% reduction in PASI from the start of methotrexate therapy; and (ii) ‘nonresponders’––those patients who showed no clear improvement, i.e. < 50% improvement in PASI while on therapy. In cases where the PASI score was not recorded, an explicit statement of response to therapy recorded in the clinical records was acceptable.

### Evaluation of toxicity

The adverse effects of methotrexate were assessed by clinical records:

#### Hepatotoxicity

Alanine transaminase (ALT) three times the upper limit of normal on two consecutive outpatient visits and/or serum level of aminoterminal of type III procollagen (P3NP) elevated above 4·2 μg L^−1^ on three consecutive occasions or 8·0 μg L^−1^ on one occasion and/or liver biopsy changes consistent with methotrexate hepatotoxicity within 3 years of starting therapy.

#### Gastrointestinal

Severe nausea, vomiting or diarrhoea, which required cessation of methotrexate or the addition of an antiemetic.

### Bioinformatics

SNPS were located and downloaded from the public HAPMAP Phase II, October 2005 database (http://www.hapmap.org/). Haplotype-tagging SNPs (*n*=47; *r*^2^ threshold of 0·8) were identified and filtered through the Haploview software omitting all markers having a minor allele frequency < 5%. Additional SNPs previously studied in *MTHFR* (rs1801133/677C>T and rs1801131/298A>C) and *ATIC* (rs2372536/347C>G) were also genotyped.

### Genotype analysis

DNA was extracted from white blood cells using a standard phenol–chloroform extraction procedure. Fifty SNPs were genotyped using Sequenom® MassARRAY™ (Sequenom Inc., San Diego, CA, U.S.A.) technology.

### Statistical analyses

The statistical software package STATA v. 8.2 (StataCorp LP, College Station, TX, U.S.A.) was used to compare genotype frequencies (χ^2^ test for trend) in order to evaluate the association of each SNP with each defined treatment outcome. Logistic regression analysis was performed using the software PLINK^15^ to adjust for age of onset of psoriasis, sex, race and, in 294 of the 374 patients, the presence or absence of folic acid supplementation.

## Results

Patients with chronic plaque psoriasis (*n*=374, males 61%, mean age of onset 24 years) treated with methotrexate were recruited into the study. Ninety-four per cent of the cohort was Caucasian and 6% either Chinese or Asian. Folic acid supplementation was being taken by 194 patients with psoriasis; 100 patients received no folate supplementation and data were unavailable on folic acid use in 80 cases.

Analysis of treatment efficacy was based on 330 patients who had completed at least 3 months of methotrexate therapy and met the criteria for either ‘responders’ (250 patients) or ‘nonresponders’ (80 patients). Adverse events had occurred in 177 patients while on methotrexate; 189 patients had ‘no adverse event’. Subgroup analysis of toxicity was recorded in 283 patients for hepatotoxicity and in 288 patients for gastrointestinal toxicity. Hepatotoxicity and gastrointestinal toxicity occurred in 65 and 79 patients, respectively.

Data were obtained for 50 SNPs in the cohort with a genotype success rate of > 90%.

### *FPGS* and *GGH*

No significant (*P*<0·05) genotypic associations were detected between SNPs in *FPGS* or *GGH* and either the efficacy or toxicity of methotrexate. After adjusting for age, sex and ethnicity the results remained nonsignificant for all SNPs other than rs10987746 in *FPGS* (adjusted *P*=0·01). No significant associations were detected upon adjustment for folic acid supplementation.

### MTHFR

No significant genotypic associations were found between SNPs in *MTHFR* and either efficacy or toxicity of methotrexate before or after adjustment for age, sex, ethnicity and folic acid. Genotypic data for the previously investigated SNPs rs1801133 and rs1801131 are shown in Table 2. Combined analysis was performed for SNP rs1801133 T allele and SNP rs1801131 C allele with no significant (*P*>0·1) association found.

**Table 2:** Genotypic associations and odds ratios (OR) for the carriage of allele 2 [with 95% confidence interval (CI)] for previously investigated single nucleotide polymorphisms in the genes methylenetetrahydrofolate reductase (*MTHFR*) and 5-aminoimidazole-4-carboxamide ribonucleotide transformylase (*ATIC*) and their association with the efficacy and toxicity of methotrexate in our psoriasis cohort

	Genotype frequencies (%)							
			
	Efficacy/**toxicity**			No efficacy/**no toxicity**				
				
Gene/ Rs number	1_1	1_2	2_2	1_1	1_2	2_2	Trend test, *P*-value	Carriage of allele 2 OR (95% CI)
*MTHFR*								
rs1801133	115 (48)	100 (42)	23 (10)	32 (42)	37 (49)	7 (9)	0·5	0·8 (0·4–1·4)
rs1801131	118 (51)	86 (37)	28 (12)	40 (54)	28 (38)	6 (8)	0·4	1·1 (0·7–2·0)
*MTHFR*								
**rs1801133**	**75 (47)**	**67 (42)**	**18 (11)**	**84 (47)**	**77 (43)**	**19 (10)**	**0·9**	**0·9 (0·6–1·6)**
**rs1801131**	**82 (53)**	**55 (34)**	**18 (13)**	**88 (50)**	**71 (40)**	**17 (10)**	**0·9**	**0·9 (0·6–1·4)**
*ATIC*								
**rs2372536**	**77 (52)**	**57 (38)**	**15 (10)**	**65 (37)**	**84 (48)**	**25 (15)**	**0·01**	**0·6 (0·3–0·9)**
**rs4672768^a^**	**76 (51)**	**57 (38)**	**16 (11)**	**65 (37)**	**84 (48)**	**25 (15)**	**0·02**	**0·6 (0·4–0·9)**

a No known function but has a borderline association with the onset of toxicity. 1, Major allele; 2, minor allele. Bold font indicates association with toxicity.

### ATIC

No significant genotypic associations were detected between SNPs in *ATIC* and the efficacy of methotrexate. However, two of the 15 SNPs were significantly (*P*<0·05) associated with onset of toxicity (Table 2). The most significant of these is the coding SNP rs2372536, with a χ^2^ test for trend of *P*=0·01. These two SNPs have a very high degree of correlation with an *r*^2^ of 1 and remain associated with onset of toxicity (*P*=0·01) after adjustment for the variables age, sex and ethnicity. A further SNP, rs4672768 was associated (*P*=0·01) with the onset of toxicity after adjustment for the above three variables. No associations remained after adjusting for folic acid supplementation. Following correction for the multiple statistical tests (Bonferroni correction factor, *n*=50) these associations became nonsignificant (*P* > 0·05). These SNPs were not associated with either hepatic or gastrointestinal toxicity (data not shown).

## Discussion

In this study, we found no significant (*P*<0·05) associations between SNPs in *FPGS*, *GGH* and *MTHFR* and either methotrexate efficacy or toxicity in patients with psoriasis. Borderline associations (*P*>0·01 to ≤ 0·05) were detected between methotrexate toxicity and two SNPs in *ATIC*. Multivariate analysis accounting for age at onset of psoriasis, sex and ethnicity showed four borderline associations between three SNPs in *ATIC* and one in *FPGS* and methotrexate toxicity. When the Bonferroni correction was performed or when analysing the subgroups of hepatotoxicity and gastrointestinal toxicity, these associations became nonsignificant (*P*>0·05). Adjustment for the supplementation with folic acid revealed no associations between any SNPs and outcomes to methotrexate treatment. We were unable to replicate any of the previously reported associations in patients with rheumatoid arthritis and two functional SNPs in *MTHFR* and one functional SNP in *ATIC*.^1^,^2^,^4^,^6^,^10^,^11^ No associations were detected for carriage of combinations of previously described functionally independent risk alleles in the genes *MTHFR* (rs1801133 T allele and rs1801131 C allele) and *ATIC* (rs2372536 G allele).

Clearly there are important limitations to the current study. Firstly, it was performed in a retrospective cohort of patients therefore not allowing a systematic, prospective and objective collection of phenotypic data. Furthermore, a diverse range of endpoints was used in studies reported in the literature which makes direct comparison difficult, particularly as the majority were performed in patients with rheumatoid arthritis. It is possible that we may have missed genetic variation across each of the genes studied which could influence response to methotrexate treatment in psoriasis patients. However, we estimate that we had 80% gene coverage of all SNPs reported on the phase II HAPMAP data.

We conclude that genetic variation across the genes *FPGS*, *GGH*, *MTHFR* and *ATIC* is not predictive of either efficacy or toxicity of methotrexate in patients with psoriasis.
